# Knowledge, attitudes and prevalence of tobacco use among physicians and dentists in Oman

**DOI:** 10.4103/0256-4947.51803

**Published:** 2009

**Authors:** Jawad A. Al-Lawati, Shalini C. Nooyi, Alya M. Al-Lawati

**Affiliations:** a Department of Non-communicable Diseases Surveillance & Control, Ministry of Health, Muscat, Oman; b Al-Nahda Hospital, Ministry of Health, Muscat, Oman

## Abstract

Tobacco use among Omani physicians and dentists has not been studied, so we conducted a cross-sectional survey using a WHO questionnaire to measure prevalence and to learn about smoking practices among this population and about their knowledge and attitudes of the health effects of tobacco use and tobacco control. The 1191 subjects who participated (787 men and 404 women) ranged in age from 24 to 65 years with a mean (SD) of 41.7 (6.8) years for men and 38.1 (6.9) years for women. The prevalence of tobacco use was 16.4% among males and less than 1% among females. Manufactured cigarettes were the most common form of tobacco used (14.7%), followed by smokeless tobacco (2.2%) and waterpipes (1.7%). Tobacco users were significantly less favorable to strict control and policy measures than never tobacco users and had less knowledge of some of the heatlh effects of tobacco use. Tobacco use among physicians and dentists in Oman is lower than in other countries in the region, but remains a cause of concern. Programs and policies should strive to maintain the low level of tobacco use or reduce it further.

Tobacco is the second major cause of death and fourth most common risk factor for disease worldwide.^1^ Health professionals often fall victim to this addiction even before embarking on a career in health care. Since tobacco use among Omani physicians and dentists has not been studied, we conducted a cross-sectional study to measure prevalence and gathered information on smoking practices and knowledge and attitudes on the health effects of tobacco use and tobacco control.

## SUBJECTS AND METHODS

The target group for this survey was all physicians and dentists employed by the Ministry of Health in Oman. The sample size was calculated based on the prevalence of smoking in the general population of 24% in males and 2% in females.^2^ Considering a non-response rate of 30% and a precision of 8% on either side of the 95% confidence interval for any point estimate, the sample size was estimated to be 1231 males and 769 females (Epinfo version 6; CDC, Atlanta, GA, USA). The questionnaire, The World Health Organization Global Health Professional Survey (see Appendix), was self-administered anonymously by each selected physician and dentist. Knowledge, attitudes and cessation counseling practices were assessed on an ordinal 5-points Likert scale where each response was ranked by giving equally spaced weight: 1, strongly agree; 2, agree; 3, unsure; 4, disagree; 5, strongly disagree. Prevalence estimates were calculated using Intercooled Stata software (version 9.1, Stata Corporation, College Station, Texas, USA, *http://www.stata.com*). Differences between mean responses of never and ever tobacco users were calculated using two independent samples *t* test. The closer the mean score of all the respondents to one, the stronger their agreement with the proposed statement. Differences were considered to be statistically significant if the *P* value was <.05.

A daily tobacco user was defined as someone who used tobacco products at the time of the survey at least once a day. An occasional smoker was someone who smoked at the time of the survey but not every day. Both of these categories were combined to give “current smoke”. When other forms of tobacco use were added, the category was labelled as “current tobacco users”. An ex-smoker was someone who smoked daily or occasionally or someone who smoked at least 100 cigarettes in his lifetime, but did not smoke at the time of the survey. A never-smoker was someone who never smoked at all or had never been a daily smoker or had smoked less than 100 cigarettes up to the time of the survey. Permission to conduct the study was obtained from the research and ethical committee of the Ministry of Health of Oman.

## RESULTS

Of the 2021 physicians and dentists invited to participate, 119 (58 men and 61 women) were found to have resigned and left the country. Of the remaining 1902 physicians and dentists, data from 1191 subjects were analyzed (787 men and 404 women). The number of subjects was lower than the calculated sample size due to the moderate response rate. The response rate was higher in men (67.1%) than in women (55.4%). Participant age ranged from 24 to 65 years. The mean (SD) age was 41.7 (6.8) years for men and 38.1 (6.9) years for women. Over 84% of physicians (n=1079) and dentists (n=48) were aged between 30-49 years. Eleven percent were Omani nationals, 29% were general practitioners, 36% were internists, 28% surgeons, 4% dentists and 2.2% were laboratory physicians.

Tobacco use was more prevalent among non-Omani males aged 30-49 years. Overall, 16.4% (129/787) of males and less than 1% (3/404) of females were current users of any form of tobacco (Table 1). The prevalence of current tobacco use among Omani males was 11% (8/73) compared to 17% (121/714) in non-Omanis (*P*=0.188). Manufactured cigarettes were the most common form of tobacco used by males (14.7%) (116/787) followed by smokeless tobacco (nasal snuff, oral snuff and chewing tobacco and betel quid) (2.2%) (17/787) and waterpipes (1.7%) (13/787).

**Table 1:** Current and ex-smokers by type of tobacco used, and specialty among physicians and dentists (n=1191) in Oman, 2001.

	Omani (n=134)		Non-Omani (n=1057)		
	Male (n=73)	Female (n=61)	Male (n=714)	Female (n=343)	Total
**All types of tobacco**					
Current users	8 (11.0)	1 (1.6)	121 (17.0)	2 (0.6)	132 (11.1)
Ex-users	6 (8.2)	0 (0.0)	125 (17.5)	4 (1.2)	135 (11.3)

**Cigarettes**					
Current users	8 (11.0)	0 (0.0)	108 (15.1)	2 (0.6)	118 (9.9)
Ex-users	6 (8.2)	0 (0.0)	136 (19.0)	4 (1.2)	146 (12.3)

**Waterpipes**					
Current users	1 (1.4)	1 (1.6)	12 (1.7)	0 (0.0)	14 (1.2)

**Smokeless tobacco**					
Current user	0 (0.0)	1 (1.6)	17 (2.4)	1 (0.3)	19 (1.6)

**Specialty**					
General practicioners (n=326)	4 (18.2)	1 (4.0)	66 (35.3)	2 (2.1)	73 (22.4)
Medical (n=411)	4 (16.0)	0 (0.0)	87 (31.0)	3 (3.2)	94 (22.9)
Surgical (n=317)	4 (26.7)	0 (0.0)	66 (36.5)	0 (0.0)	70 (22.1)
Laboratory** (n=25)	0 (0.0)	0 (0.0)	3 (42.8)	0 (0.0)	3 (12.0)
Dental (n=48)	1 (20.0)	0 (0.0)	11 (42.3)	0 (0.0)	12 (25.0)

Values are number of subjects and percentage of column total for type of tobacco use or total for each specialty. Note: In 3 subjects the nationality was unknown and they were excluded in the above analysis. In 64 subjects the specialty was unknown but they were included in the overall analysis. No attempt was made to identify ex-users of waterpipes and smokeless tobacco. *Laboratory physicians e.g. pathologists or microbiologists.

There were statistically significant differences in physician and dentist perception of clinical practice and attitudes towards tobacco control polices (Table 2). Tobacco users were significantly less favorable to strict control and policy measures than never tobacco users and had less knowledge of some of the health effects of tobacco use.

**Table 2:** Comparison of knowledge and attitudes of physicians and dentists towards tobacco control, Oman, 2001.

	Mean score		
Statement	Never used tobacco	Ever used tobacco	Pvalue
Patient's chances to quit are increased if a health professional advises them so	1.9	2.1	.001
Health professionals should routinely ask about their patients smoking habits.	1.6	1.7	.046
Heath professionals should routinely advise their smoking patients to quit smoking.	1.5	1.6	.004
Health professionals who smoke are less likely to advise people to stop smoking.	2.1	2.7	.001
Health professionals should get specific training on cessation techniques.	1.7	1.9	.002
Smoking in enclosed public places should be prohibited.	1.1	1.3	.001
Health warnings on cigarette packages should be in big print.	1.3	1.5	.001
Tobacco sales should be banned to children and adolescents.	1.1	1.2	.092
Sport sponsorships by tobacco industry should be banned.	1.5	1.6	.390
There should be a complete ban on the advertising of tobacco products.	1.5	1.7	.019
Hospitals and health care centers should be “smoke-free”.	1.1	1.2	.007
The price of tobacco products should be increased sharply.	1.7	2.2	.001
Maternal smoking during pregnancy increases the risk of SiDS*	1.7	1.9	.012
Passive smoking increases the risk of lung disease in non-smoking adults.	1.5	1.7	.001
Passive smoking increases the risk of heart disease in non-smoking adults.	1.8	1.9	.104
Paternal smoking increases the risk of lower respiratory tract illnesses such as pneumonia in exposed children.	1.8	1.9	.015

* SIDS, Sudden infant death syndrome

## DISCUSSION

This study documents for the first time, the prevalence of tobacco use, knowledge of and attitudes towards tobacco control among physicians and dentists working in Oman. The study shows that tobacco use is predominantly a male disease and that its use among Omani physicians and dentists is relatively low (11%) compared to the general population (24%).^2^ In 1995, Sulaiman et al reported that the prevalence of any current tobacco use among Omanis aged 15 years and above, in the general population, was 15.5% in males and 1.5% in females.^3^ A second community-based survey (in 2000) among Omanis aged 20 and above, reported similar figures for current smokers (13.4% males and 0.5% females).^4^

Using an identical questionnaire and methodology, higher rates of tobacco use were reported among physicians and dentists from other Arab countries such as the United Arab Emirates (43%), Kuwait (31%), Bahrain (23%), Libya (17.2%), Saudi Arabia (14.2%) and Qatar (12%).^5^–^7^ The low prevalence rates of tobacco use in Oman have been a consistent finding when compared to other Arabian Gulf states. This may be attributed to the fact that Oman's renaissance, which opened it to world markets, including the tobacco trade, occurred at least a decade later (in the 1970s following commercial exploitation of oil and a regime change) than other Gulf states. Further, tobacco use has been traditionally a taboo in Oman's non-coastal areas, in congruence with the strict ruling by the religious authorities (the Abadhi sect of Islam in Oman) that have long declared tobacco use as haram (religiously forbidden).

Although waterpipe smoking is emerging as a fashionable mode of tobacco use in the Arab world,^8^ its use among physicians and dentists in Oman was extremely rare (1.2%). This could be because it is not so easy to use (compared to smoking cigarettes). Media advertisement for cigarettes could also have tipped the balance in favor of cigarettes considering there are none or rarely any advertisements for waterpipes.

Our results show a high prevalence of tobacco use among dentists. While this could be due to the small sample size of this group, other studies have also shown a higher prevalence of tobacco use among dentists compared to physicians of other specialties.^9^,^10^ In addition, the percentage of Omani doctors and dentists in the Ministry of Health is about 24% while in our study it was 10% because we selected a sample from all males disregarding the stratification of Omanis and non-Omanis. For females, the calculated sample size was nearly the same as the total number of female dentists and doctors working in the Ministry of Health.

Physicians and dentists, who had used or currently use tobacco, were less approving of evidence-based measures to control tobacco than never users. This finding is consistent with other studies where smokers report less support for tobacco control measures than nontsmokers.^11^ However, it must be cautioned that not all smokers are homogeneously opposed to all tobacco control polices.^12^

There were a few limitations to this study. The low response rate among female physicians and dentists could be attributed to the social taboo and unacceptability of tobacco use among women in oriental societies, including Omanis. Low female participation has also been reported from similar studies across other Arab nations (Jordan 13%, Saudi Arabia 21%, Egypt 24%, Qatar 28%, Libya 41%) compared to 34% in Oman.^13^ Our survey has excluded physicians and dentists working in police and army hospitals as well as private sector clinics. We estimated this group to be around 25% to 30% of all the physicians and dentists working in the country.

We conclude that tobacco use among physicians is a cause for concern, more so among non-national physicians and dentists than among Omanis. Efforts should capitalize on this finding and programs and polices should strive to maintain this low level of tobacco use or reduce it even further.

**Appendix:**
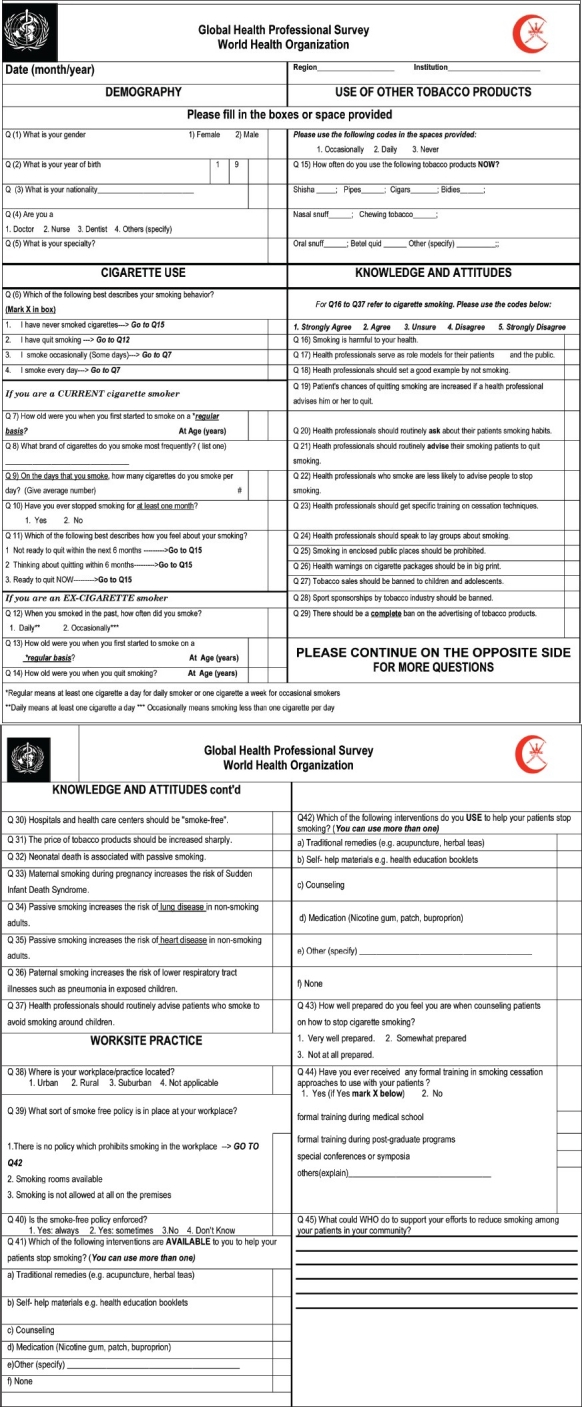
World Health organization Global Health Professional Survey.
